# Porous Si Partially Filled with Water Molecules—Crystal Structure, Energy Bands and Optical Properties from First Principles

**DOI:** 10.3390/nano10020396

**Published:** 2020-02-24

**Authors:** Ya. Shchur, O. Pavlyuk, A.S. Andrushchak, S. Vitusevich, A.V. Kityk

**Affiliations:** 1 Institute for Condensed Matter Physics, 1 Svientsitskii str., 79011 Lviv, Ukraine; 2 Department of Inorganic Chemistry, Faculty of Chemistry, Ivan Franko National University of Lviv, 6 Kyryla and Mefodia str., 79005 Lviv, Ukraine; pavalex@gmail.com; 3 Department of Applied Physics and Nanomaterials Science, Lviv Polytechnic National University, 12 S. Bandery str., 79013 Lviv, Ukraine; anatolii.s.andrushchak@lpnu.ua; 4 Institute of Bioelectronics (IBI-3), Forschungszentrum Juelich, D-52425 Juelich, Germany; s.vitusevich@fz-juelich.de; 5 Faculty of the Electrical Engineering, Czestochowa University of Technology, Al. Armii Krajowej 17, 42-200 Czestochowa, Poland; andriy.kityk@univie.ac.at

**Keywords:** porous silicon, hydrogen bond, density functional theory, energy bands, refractive index, extinction coefficient, 71.15.Mb, 73.20.At, 77.84.Fa, 78.67.Bf, 81.07.Gf

## Abstract

The paper reports the results on first-principles investigation of energy band spectrum and optical properties of bulk and nanoporous silicon. We present the evolution of energy band-gap, refractive indices and extinction coefficients going from the bulk Si of cubic symmetry to porous Si with periodically ordered square-shaped pores of 7.34, 11.26 and 15.40 Å width. We consider two natural processes observed in practice, the hydroxylation of Si pores (introduction of OH groups into pores) and the penetration of water molecules into Si pores, as well as their impact on the electronic spectrum and optical properties of Si superstructures. The penetration of OH groups into the pores of the smallest 7.34 Å width causes a disintegration of hydroxyl groups and forms non-bonded protons which might be a reason for proton conductivity of porous Si. The porosity of silicon increases the extinction coefficient, *k*, in the visible range of the spectrum. The water structuring in pores of various diameters is analysed in detail. By using the bond valence sum approach we demonstrate that the types and geometry of most of hydrogen bonds created within the pores manifest a structural evolution from distorted hydrogen bonds inherent to small pores (∼7 Å) to typical hydrogen bonds observed by us in larger pores (∼15 Å) which are consistent with those observed in a wide database of inorganic crystals.

## 1. Introduction

Silicon, Si, has been one of the most important technological materials for the past decades. The main physical properties of bulk silicon are well known being widely used in technology. The bulk silicon is a diamond type crystal of cubic symmetry. Another morphological form of silicon, porous silicon, characterized by a set of elongated pores of various sizes and shapes is a macroscopically anisotropic material. The range of pore widths is very broad comprising the micropores (<2 nm), mesopores (2–50 nm) and macropores (>50 nm) depending on bulk Si substrate and electrochemical etching conditions [1]. The physical properties of porous Si have been intensively studied since the discovery of its photoluminescence in the red visible range at room temperature in 1990 [1]. To date, there exists a voluminous scientific literature devoted to the investigation of physical properties and technological applications of porous Si-based materials. Referring to the comprehensive reviews formerly published by Feng and Tsu [2], Cullis, Canham and Calcott [3], Bisi, Ossicini and Pavesi [4] and Koshida and Nakamura [5], one may point out here only some certain structural [6,7], Raman scattering [8], optical [9], elipsometric [10,11] and electrical transport [12,13] methods. The technological importance of porous Si has been mainly related to its visible range luminescence. The tuning of photoluminescence of Si quantum dots present in porous Si in the spectral region from the near infrared to the ultraviolet due to the quantum confinement and surface passivation was revealed in Reference [14]. Owing to a large surface area of the porous Si and consequently, due to its high capacity, the porous Si is considered as a promising material for lithium-ion battery anodes [15]. Moreover, porous Si showed to good advantage as a very promising biomaterial due to its biocompatibility, bioresorbability, and degradability. In microbiological engineering, the porous Si is mainly used as a cell scaffold tool [16]. In life science, nanocomposites based on Co nanoparticles embedded into a porous Si matrix manifested a great potential as biomarkers [17].

To get a comprehensive understanding of underlying physical phenomena, the porous Si has been intensively studied using various theoretical methods, namely by model calculations [18,19], effective mass approximation [20], tight binding [21,22], density functional theory [23,24,25,26].

It is difficult to overstate the role and significance of hydrogen bonding in chemistry, physics, material science and biology. Although the term “hydrogen bond” has been used in the scientific literature for more than a hundred years [27], the discussions regarding the nature and features of this type of interatomic binding have not ended yet [28]. Regardless of some theoretical issues that are still debated, the hydrogen bonding plays a substantial role in assembling of many liquids, amorphous solids and crystals of most organic [29] and numerous inorganic compounds [30]. Proton ordering in hydrogen O-H···O bonds plays a crucial role in structural phase transitions in many hydrogen bonded crystals, for example, in the crystals of KH${}_{2}$PO${}_{4}$ type [31,32,33,34,35]. It turned out that hydrogen bonds may involve the metal ions, particularly the transition metals [36]. In general sense, hydrogen bonds play an important role in the existence of all living organisms without exception, because, in a hierarchy of chemical interactions, only hydrogen bonds are capable of moderating the low-energy, directional interactions needed for the operation of genetic and catalytic tasks [37].

Despite the intensive experimental and theoretical investigations of porous Si mentioned above, there are still a lot of gaps of knowledge to be properly filled out. The goal of our study is twofold. Firstly, we are aimed at getting information at the first-principles level regarding the evolution of the energy band structure and optical properties starting from the bulk homogeneous silicon and going to its porous morphological forms. Performing our research we took into account the processes inherent to real porous materials, that is, the penetration of hydroxyl OH groups and water inside the pores from the ambient atmosphere. The dynamics of hydrogen atoms and hydrogen bonds of water confined in the silica gel pores of ∼2 nm diameter was recently reported [38]. However, precise morphology and type of water structuring within nanosized pores of diameter less than 2 nm remains an open question. The second purpose of our study is to shed light on water structuring within the small periodically ordered voids and, more generally, on the peculiarities of hydrogen bonds confined within ∼1 nm periodic pores. Particularly, we are interested in monitoring the transformation of hydrogen bond geometry at changing the Si pore diameter.

Physical anisotropy of porous Si opens up broad prospects for many new practical applications of this material especially keeping in mind the conceivable possibilities of filling the pores with various substances. Tailorable pore size and/or porosity of nanoporous Si enables the modification of its dielectric and optical properties, for example, optical birefringence, or tuning the photonic and electronic properties, such as band-gap energy and its type (*direct* or *indirect*) which is also relevant to its optical absorption or luminescence emission characteristics. In a general sense, our compound may be treated as a nanocomposite material composed of porous Si, hydroxyl groups and water molecules embedded within the pores. Hence, any constituents, OH groups or H${}_{2}$O molecules, influence the energy band spectrum and the optical properties of porous Si. What exactly is the quantitative impact of these entities is another motivation for our research.

## 2. Computational Details

Our calculations were performed within the density functional perturbation approach using the generalized gradient approximation (GGA) [39] in Perdew-Burke-Ernzerhof parameterization [40] with taking into account the long-range dispersion effects as suggested by Grimme et al. (DFT-D3 method) [41]. The *ab initio* package ABINIT [42,43] was utilized in our study. The norm-conserving ONCVPSP pseudopotentials were used with Si(3s${}^{2}$3p${}^{2}$), O(2s${}^{2}$2p${}^{4}$) and H(1s${}^{1}$) valence states. The convergence analysis was performed concerning both the sampling of the Brillouin zone using the Monkhorst-Pack scheme [44] and the kinetic energy cut-off for plane-wave calculations. We chose the 4 × 4 × 2 grid for supercells with a comparatively small number of atoms, less than 100. To maintain a reasonable balance between the computing time and the numerical accuracy of our data, we decreased the Brillouin zone grid to 2 × 2 × 2 value for all supercells containing more than 100 atoms (see Table 1).

Structural optimization was done within the Broyden-Fletcher-Goldfarb-Shanno algorithm [46]. In the worst case of the largest 6aSi supercell, the maximal forces acting on each atom were reduced to the value lower than 1.3 × 10${}^{-4}$ eV/Å, whereas for all other structures considered in our work these forces were reduced to much lower values (see Table 1). For the cut-off energy, E${}_{cut}$, we used the same value of 1632 eV with the cut-off smearing of 13.6 eV for all structures considered here.

## 3. Space Morphology and Stability Conditions

**Figure 1:**
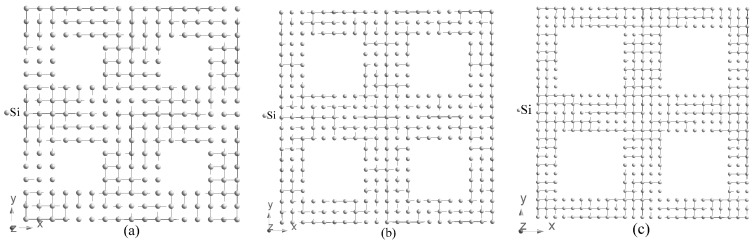
Structure of porous Si, 4aSi (4a*4a*c) (**a**), 5aSi (5a*5a*c) (**b**), 6aSi (6a*6a*c) (**c**). Each figure contains four supercells.

Bulk silicon, Si, has a cubic crystal structure of diamond type ($Fd\bar{3}m$ space group, #227, a${}_{cubic}$ = 5.4331 Å). In the present work, we investigate porous Si with periodically arranged square shaped through pores. In this case, the cubic symmetry of bulk silicon is lowered to the tetrahedral one ($P\bar{4}m2$, #115, a = b = 3.8403, c = 5.4310 Å) which is a subgroup of $Fd\bar{3}m$ space group. Three tetrahedral supercells of porous silicon with periodic boundary conditions are considered in our paper, that is, 4a*4a*c (hereafter abbreviated as 4aSi), 5a*5a*c (5aSi) and 6a*6a*c (6aSi) with the following corresponding size of pores 7.34, 11.26 and 15.40 Å, respectively (see Figure 1). Note that a and c correspond to lattice parameters of bulk Si in the tetrahedral setting.

The real microporous Si (pores < 2 nm) consists of the net of irregular pores of various shapes, sizes and inter-pore walls. In order to simulate the electronic properties of this material by first-principles methods that use periodic boundary conditions, we need to consider the periodic sequence of the same pores. To simplify the transition from the cubic structure inherent to the bulk Si to tetrahedrally coordinated pores, we decided to use the square-shaped voids. Placing the additional silicon ions into the corners of square-shaped pores of the smallest 7.34 Å diameter led to a stairs-like pore with the shape far from both circle and square. To preserve the uniformity of our consideration, we operated with square-shaped voids also for all higher dimensionality supercells. Note that the square-shaped void of 5 ∗ 5 μm is a quite typical geometry for macroporous Si (>50 nm) used for the fabrication of photonic crystals [47]. Recently, the square-shaped 400 ∗ 400 nm Si pores were successfully realized for nucleation of sub-micrometer protein crystals in a silicon mesh [48]. In our opinion, the shape of a pore plays a relatively minor role compared with pore diameter, supercell dimension and porosity of porous Si.

**Figure 2:**
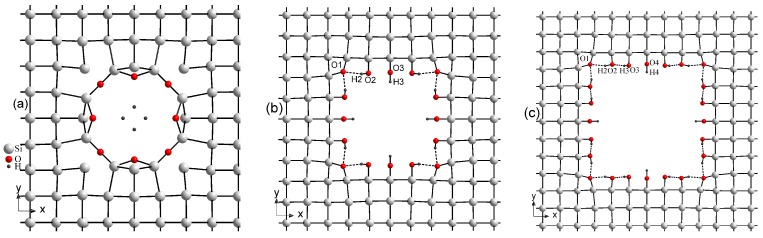
Supercells of porous Si decorated with OH hydroxyl groups, 4aSi+OH (**a**), 5aSi+OH (**b**), 6aSi+OH (**c**).

Due to the high chemical activity of hydroxyl OH groups, the pores of porous Si are normally filled with OH radicals that are present in the air at ambient conditions. We took into consideration this phenomenon in our calculations. Figure 2 presents the optimized crystal structure of three various supercells of porous Si decorated with hydroxyl groups. As seen in Figure 2b,c, in two supercells 5aSi and 6aSi, OH groups create a certain covalent bonding with the Si atoms deposited on the surfaces of pores. However, in the case of the smallest 4aSi supercell, the OH radicals turn out to be destroyed forming some amount of almost free hydrogen atoms shifted to the center of nanopore (Figure 2a). Both oxygen atoms coordinate with two Si atoms: the first has a linear coordination environment (R(O-Si) = 1.66 Å), the second lies in position with angular neighbor location (R(O-Si) = 1.74 Å, *∠*O-Si-O - 115.0°). Optimized interatomic O-H distances are in the range of 2.24–3.01 Å whereas the H-H distances turn out to be even smaller, i.e., 1.7 and 1.66 Å. For comparison, the inter-hydrogen distance in molecular hydrogen is ∼0.74 Å and in the molecular ion H${}_{2}^{+}$ is 1.06 Å [49]. From the crystal-chemical point of view, such a coordination geometry of hydrogen atoms seems to be rather problematic for practical implementation, though it explains a metal-like conductive behavior of this 4aSi structure that will be discussed below.

The next level of our modeling was embedding the water molecules inside the pores already decorated with hydroxyl OH groups. We dealt with a comparatively small number of H${}_{2}$O molecules in pores. Choosing a particular number of water molecules, we aimed at keeping a reasonable balance between, on the one hand, the reliability of the physical phenomenon of water penetration into the Si pores due to the air humidity and the available computing resources, on the other hand. The water has a definite impact on the energy band spectrum of porous Si. One may come to this conclusion by comparing the band-gap energies for the same 5aSi supercell filled with the different number of water molecules, namely E${}_{g}$(5aSi+8H${}_{2}$O) = 0.809 eV and E${}_{g}$(5aSi+16H${}_{2}$O) = 0.766 eV (see Table 1). However, the precise quantitative expressions showing how a certain number of water molecules influences the electronic band spectrum for certain Si supercells could not be realized within our research because of the considerable computing time needed to accomplish this task.

**Figure 3:**
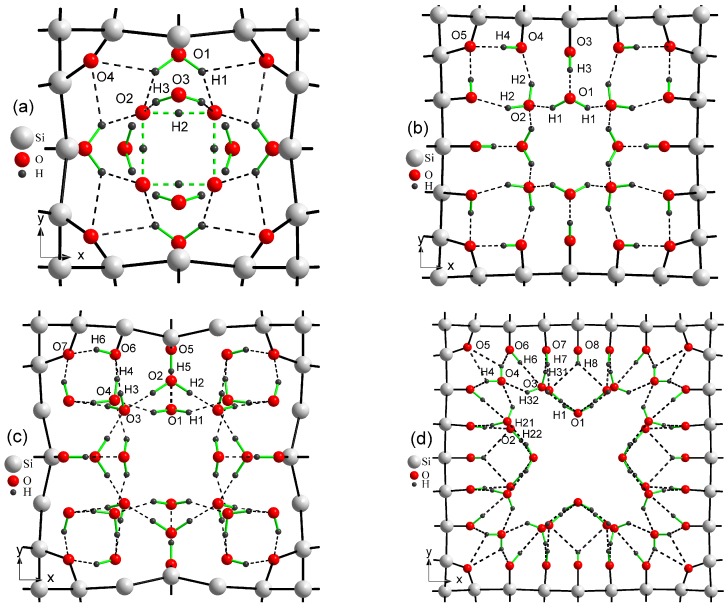
Pores of Si filled with molecules of water. 4aSi+8H${}_{2}$O (**a**), 5aSi+8H${}_{2}$O (**b**), 5aSi+16H${}_{2}$O (**c**), 6aSi+24H${}_{2}$O (**d**). Only hydrogen bonds shorter than 2.6 Å are depicted in figure by dashed lines.

Figure 3 depicts four arrangements of water molecules in porous Si selected by us for further consideration. The first and fourth cases, Figure 3a,d, correspond to the smallest and largest pores, 4aSi and 6aSi, filled with 8 and 24 H_2_O molecules, respectively. The second and third arrangements, Figure 3b,c, represent the middle pore structure, 5aSi, filled with 8 and 16 molecules of water, respectively. As seen in this Figure, an increase of water molecules in the same supercell, 5aSi, causes a complete reconstruction of water arrangement. Consequently, the whole net of hydrogen bonds, undergoes a significant rearrangement which will be discussed below. Note that tetrahedral symmetry is inherent to the whole set of hydroxyl groups, water molecules and hydrogen bonds of all superstructures considered in our work.

It turns out that the geometry of H${}_{2}$O molecules greatly depends on the size of the pores. In Table 2 we list the O-H lengths and O-H-O angles of water molecules embedded into pores. In this table, we also give the lengths of O-H acceptor, H···O donor bonds (only shorter than 2.6 Å) and *∠*O-H···O angles of hydrogen bonds O-H···O. In the gaseous phase, the mean H-O length of a water molecule is about 0.958 Å, the mean angle *∠*H-O-H = 104.47° [50]. The largest angle deviation of nearly 10° from the mean water *∠*H-O-H value is observed in H${}_{2}$-O${}_{2}$-H${}_{2}^{{}^{\prime }}$ molecule, that is, *∠*H${}_{2}$-O${}_{2}$-H${}_{2}^{{}^{\prime }}$ = 93.9°, (see Figure 3a) placed in the smallest 4aSi pores (pore size is 7.34 Å). Such a comparably large deviation may be explained by a rather peculiar crystal field created by a set of oxygen and hydrogen atoms confined within the tight pore of 7.34 Å. With increasing pore size to 11.26 and 15.40 Å of 5aSi and 6aSi structures, respectively, the geometry of water molecules tends to its typical average form (see Table 2). In 6aSi*H${}_{2}$O structure, there is a clear predominance of some stretching of water molecules regarding their typical angle *∠*H-O-H = 104.47°. The valence angles of three H${}_{2}$O molecules lie in the range of 105.0–111.4° whereas the fourth water molecule appears to be slightly compressed to the angle *∠*H-O-H = 102.4°. Two water molecules in 6aSi*H${}_{2}$O supercell, H${}_{21}$-O${}_{2}$-H${}_{22}$ and H${}_{31}$-O${}_{3}$-H${}_{32}$, demonstrate asymmetric shape with different O-H lengths within the same molecule, that is, R(O${}_{2}$-H${}_{21}$) = 0.976 Å, R(O${}_{2}$-H${}_{22}$) = 0.989 Å and R(O${}_{3}$-H${}_{31}$) = 0.981 Å, R(O${}_{3}$-H${}_{32}$) = 0.990 Å. Once again, this asymmetry may be explained by the effect of confinement inherent to small pores. The distribution of electron density within the pores is formed to a great extent by a very complicated net of O-H···O hydrogen bonds.

A complex network of hydrogen bonds created in Si pores is another peculiar feature of porous Si that deserves to be noted here. In Figure 3 we depicted only hydrogen bonds of the lengths smaller than 2.6 Å due to the baffling complexity of the total net of O-H···O bonds. According to the paper by Zhang and Xue [51] based on the analysis of 404 hydrogen bonding inorganic systems from 128 structure types, most of single hydrogen bonds and the majority of multi-furcated hydrogen bonds satisfy the criterion R(O-H···O) ≤ 2.35 Å and *∠*O-H···O ≥ 140°. Most of the hydrogen bonds listed in Table 2 match these conditions. More precisely, all R(O-H) distances calculated in the present work lie within the range 0.97–1.03 Å. Nearly 75% of hydrogen bonding systems from Zhang and Xue’s database [51] manifest similar R(O-H) values. Almost all R(H···O) distances and *∠*O-H···O angles calculated in our work are placed in the 1.57–2.26 Å and 137–179° regions, respectively, which are consistent with ∼90% of the corresponding experimentally observed values of single bonds and major components of multi-furcated hydrogen bonds [51].

**Figure 4:**
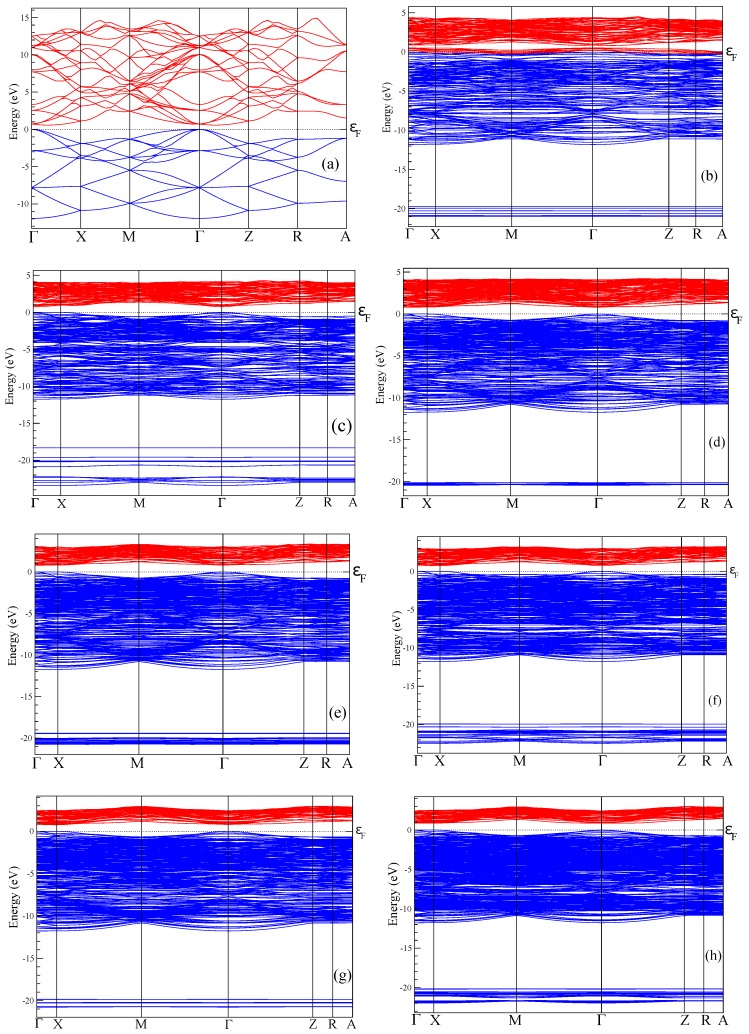
Energy bands of bulk Si (**a**) and porous Si supercells of 4aSi+OH (**b**), 4aSi+8H${}_{2}$O (**c**), 5aSi+OH (**d**), 5aSi+8H${}_{2}$O (**e**), 5aSi+16H${}_{2}$O (**f**), 6aSi+OH (**g**), 6aSi+24H${}_{2}$O (**h**).

We would like to emphasize some structural peculiarities of 4aSi+H${}_{2}$O supercell. Almost all oxygen atoms are involved in strong covalent O-Si or donor O-H chemical bonds, with the only exception of O${}_{2}$ atom which is involved in acceptor H${}_{3}$···O${}_{2}$, H${}_{1}$···O${}_{2}$ and H${}_{2}$···O${}_{2}$ bonds (Figure 3a). H${}_{2}$ atom is placed in the middle between two neighbor O${}_{2}$ atoms creating thus some unexpected kind of O${}_{2}$···H${}_{2}$···O${}_{2}$ hydrogen bond. A similar spatial structure is observed in HF${}_{2}^{-}$ ion [52]. However, the presence of symmetric hydrogen bonds has not been yet established among oxygen-containing compounds by diffraction methods. Meanwhile, this structural feature resembles the structural peculiarity observed in some hydrogen bonding crystals, for example, of KH${}_{2}$PO${}_{4}$ type in which the center location of H atoms in hydrogen bonds describes the static time-averaged picture of high-symmetry center-symmetric phase. On the dynamical scale of very short times, the H atoms make a flip-flop motion between two off-center positions in the hydrogen bond [31,32,33,34,35]. The involvement of H${}_{2}$ atom in O${}_{2}$···H${}_{2}$···O${}_{2}$ bond eliminates the metal-like conductivity inherent to 4aSi+OH supercell creating a definite energy gap E${}_{g}$ = 0.752 eV in 4aSi+H${}_{2}$O supercell (see Figure 4c). Therefore, one may come to a rather paradoxical conclusion that embedding the water molecules into 4aSi+OH pores changes its conductive properties from metal-like to insulator-like ones.

In order to check the crystal chemistry correctness of our structural modeling, we use the bond valence sum (BVS) concept [53]. According to this approach, the V${}_{H}$ valence of a hydrogen atom is the sum of the individual bond valences *v${}_{i}$* of O-H or O···H bonds surrounding a certain H hydrogen atom, that is,

$$\begin{array}{c} V_{H}=\sum_{i}v_{i}=\sum_{i}\exp\frac{R_{0}-R_{i}}{b}, \end{array}$$

where R${}_{i}$ is an observed length of *i*-th bond, R${}_{0}$ = 0.831 Å and b = 0.565 Å are the fitting constants evaluated for most of the inorganic crystals from the database analyzed in Reference [53] that satisfied the V${}_{H}$≈ 1 condition. Calculating the BVS for certain hydrogen atoms of all structures considered in our paper we took into account only those oxygen atoms that satisfy the geometrical criterion R(O···H) ≤ 3.04 Å, that is, a sum of van der Waal’s radii of oxygen. As seen in Table 2, most of V${}_{H}$ values do not exit the 1.0 ± 0.08 region. For all water-contained supercells, 4aSi+H${}_{2}$O, 5aSi+H${}_{2}$O(1), 5aSi+H${}_{2}$O(2) and 6aSi+H${}_{2}$O, there is a mean value V${}_{H}$ = 1.024 ± 0.066. This proximity to the unity of V${}_{H}$’s should testify in favor of crystal stability and the correctness of structures considered by us.

## 4. Electronic Properties

**Figure 5:**
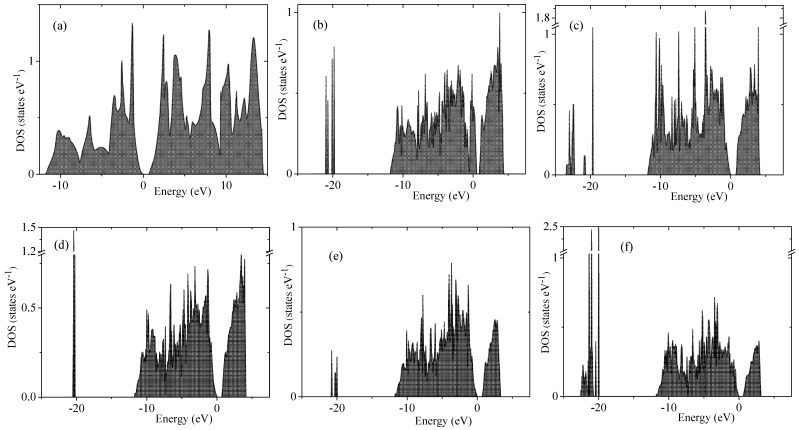
Density of states of bulk Si (**a**) and porous Si supercells of 4aSi+OH (**b**), 4aSi+8H${}_{2}$O (**c**), 5aSi+OH (**d**), 5aSi+8H${}_{2}$O (**e**), 5aSi+16H${}_{2}$O (**f**).

The electronic spectrum of porous silicon along with the density of electronic states is depicted in Figure 4 and Figure 5, respectively. The energy levels of the valence band are drawn in Figure 4 in blue color whereas the conduction band is depicted in red color. Energy band-gap of each silicon porous structure is presented in Table 1.

**Figure 6:**
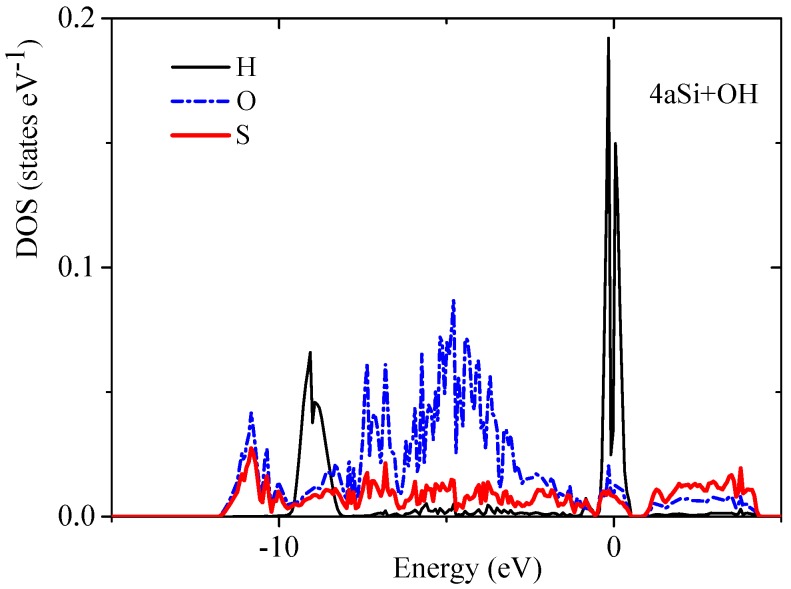
Partial DOS of hydrogen, oxygen and silicon atoms of 4aSi+OH structure.

Transformation of the space morphology from the bulk to porous form causes a splitting of the valence band into two valence sub-bands separated by a distinct energy gap. Several features merit special attention. The most peculiar feature of electronic spectra of porous Si is the absence of an energy gap between the valence and conductive bands in the spectrum of the smallest 4aSi pores containing hydroxyl OH groups (see Figure 4b). This 4aSi+OH superstructure reveals metal-like conductive properties. The probable reason is the presence of four unbounded protons in the supercell. As seen from the representative partial DOS of hydrogen, oxygen and silicon atoms of 4aSi+OH hydroxylated structure (Figure 6), the main contribution to DOS near Fermi level comes from hydrogen atoms (zero set to Fermi level). Oxygen and silicon atoms give a certain but much smaller contribution compared to hydrogen.

Four free hydrogen ions are also inherent to 4aSi+H${}_{2}$O superstructure (4aSi+OH supercell + 8 molecules of H${}_{2}$O, see Figure 3a). However, this superstructure manifests a quite distinct band gap of 0.752 eV (Figure 4c and Figure 5c). The most probable reason of this difference is the hydrogen O${}_{2}$···H${}_{3}$···O${}_{2}$ bonds which effectively tie together four H${}_{3}$ protons in 4aSi+H${}_{2}$O supercell.

**Figure 7:**
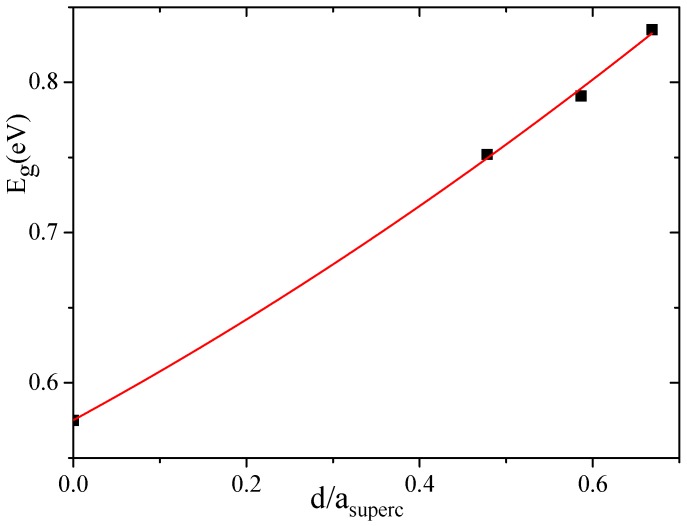
Band-gap energy as a function of the pore spacing ratio, $\frac{d}{a_{superc}}$. Average E${}_{g}$ values were used for 4aSi, 5aSi and 6aSi supercells.

Another peculiarity of the energy band spectra of all nanoporous Si materials calculated by us is the lower energy band gap compared to the experimental E${}_{g}$ value observed in the bulk Si. As indicated in Table 1, the calculated E${}_{g}^{bulk}$ = 0.575 eV of the bulk silicon is almost two times smaller than the experimental value of 1.14 eV [45]. The reason of such underestimation is the well-known DFT band-gap problem [54]. However, as follows from Table 1, the energy band gap tends to increase with an increasing pore diameter of *d*. In Figure 7, we plot the band-gap values as a function of the pore spacing ratio, $\frac{d}{a_{superc}}$, where $a_{superc}$ is a unit cell constant of supercell. In this figure, we used the average E${}_{g}$ value of all corresponding values calculated for a certain supercell, that is, of 4aSi, 5aSi and 6aSi. As seen in this figure, the energy band gap obeys non-linear law,

$$\begin{array}{c} E_{g}\left(\frac{d}{a_{superc}}\right)=E_{g}^{bulk}+0.313\left(53\right)\left(\frac{d}{a_{superc}}\right)+0.107\left(83\right){\left(\frac{d}{a_{superc}}\right)}^{2}. \end{array}$$

The increase of E${}_{g}$ with an increasing porosity does not seem strange at all because the total volume of the empty space increases with an increase of the pore diameter. Note that the porosity changes the type of the forbidden energy gap, thus changing it from an *indirect* type inherent to bulk homogeneous Si to a *direct* type detected by us in porous Si with 6aSi supercell (see Table 1).

## 5. Optical Properties

**Figure 8:**
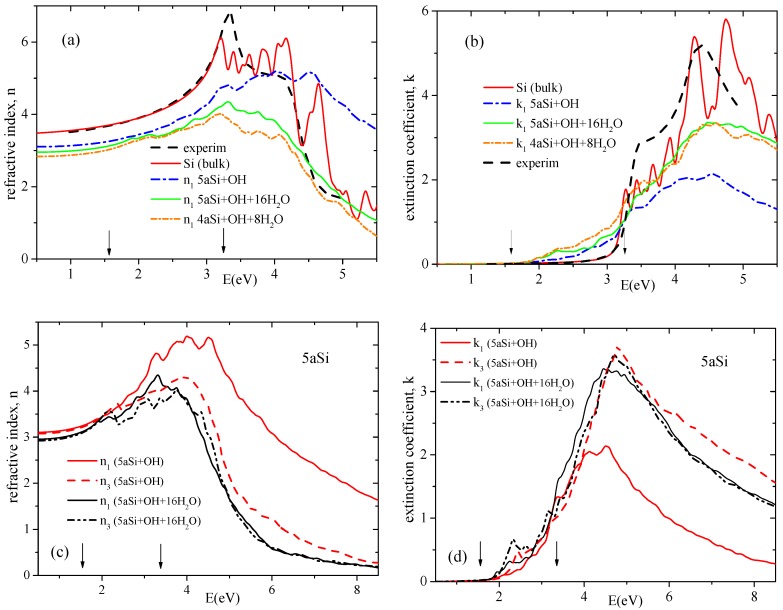
Dispersion of refractive indices *n* (**a**,**c**) and extinction coefficients *k* (**b**,**d**) of bulk and porous Si of different pore diameters. Arrows indicate the margins of the visible range. Experimental data are taken from Reference [55].

Crystalline silicon is an optically isotropic material with the same refractive index in any direction ($Fd\bar{3}m$ cubic symmetry). Porous silicon loses the cubic symmetry inherent to the bulk Si and becomes an optically anisotropic material. We have chosen the tetragonal subgroup $P\bar{4}m2$ of cubic space group for theoretical treatment of porous Si. This symmetry admits two different refractive indices (optical anisotropy) which we hereafter denote as $n_{1}$ = $n_{2}$ and $n_{3}$. Calculating the high-frequency dispersion of the complex dielectric $\varepsilon^{*}$ function and using the following relations

$$\begin{array}{cc} 2n^{2}=\sqrt{{\varepsilon^{\prime }}^{2}+{\varepsilon^{\prime \prime }}^{2}}+\varepsilon^{\prime }, & 2k^{2}=\sqrt{{\varepsilon^{\prime }}^{2}+{\varepsilon^{\prime \prime }}^{2}}-\varepsilon^{\prime }, \end{array}$$

one may easily obtain the dispersion of refractive indices $n_{1}$ and $n_{3}$ and extinction coefficients $k_{1}$ and $k_{3}$. In Figure 8 we present the dispersion of refractive indices and extinction coefficients of various Si space morphologies. As seen in Figure 8a,b, there is a very good agreement in the visible range between the calculated refractive index and the extinction coefficient of the bulk cubic Si and the experimental data [55]. Compared to the bulk silicon, the porosity of Si generally decreases the refractive indices within the whole calculated spectral range and increases the extinction coefficient *k* in the visible optical region (Figure 8a,b). The same tendency of increasing the extinction coefficient in the visible range at transition from bulk three-dimensional material to the ordered sequence of one-dimensional nano-rods was formerly observed in PbHPO${}_{4}$ crystal [56]. Moreover, the Si porosity increases the optical anisotropy ($n_{1}$ > $n_{3}$) especially in ultraviolet region and in the range higher than the near ultraviolet one (above 3.0 eV). For representative purpose in Figure 8c,d we depict only the data of 5aSi porous superstructure.

Filling the pores with water molecules causes a decrease of the $n_{1}$ refractive index to the value comparable with $n_{3}$ (see Figure 8c), thus reducing the optical birefringence.

**Figure 9:**
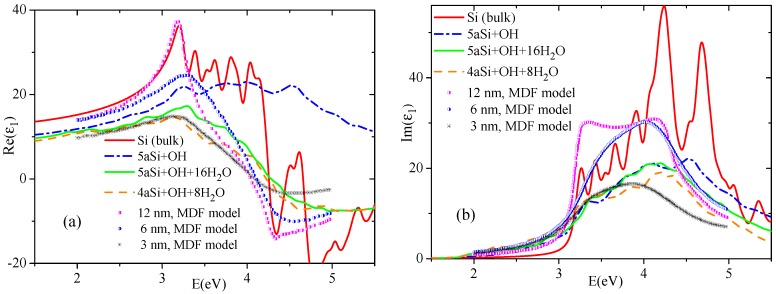
Dispersion of dielectric function, Re($\varepsilon_{1}$) (**a**) and Im($\varepsilon_{1}$) (**b**), of bulk and porous Si of 4aSi and 5aSi supercells. The data represented by symbols are reproduced from Reference [19] for porous Si films with nano-grains of different size. All lines correspond to data calculated in our paper.

It is quite instructive to compare the results of our calculations with experimental data. Formerly, the model dielectric function (MDF) approach [19] was successfully used for characterization of ellipsometric reflectivity spectra measured on porous sponge-like Si thin films with different nanocrystal (wall) sizes, i.e., 12, 6 and 3 nm. Based on the fitted parameters, the authors of Reference [19] established a high-frequency dielectric dispersion of porous Si thin films with various nano-grains, which are reproduced in Figure 9 by symbols. In this figure, we also present the dispersion of real and imaginary parts, Re($\varepsilon_{1}$) and Im($\varepsilon_{1}$), of dielectric function $\varepsilon_{1}^{*}$ of the same Si space morphologies as those depicted in Figure 8a,b. All the data simulated by us are drawn in Figure 9 by lines. As seen in this Figure, for two marginal cases, that is, for the bulk Si and for the smallest 4aSi supercell, there is a good qualitative agreement between our data and those published by Petrik et al. [19]. Real part, Re($\varepsilon_{1}$), obtained within MDF approach for 12 nm nano-sized Si crystals almost coincides in the visible optical range with our results calculated for bulk Si crystal. Imaginary part, Im($\varepsilon_{1}$), shows a worse correlation since our data present the sequence of oscillation peaks whereas the Petrik et al’s data manifest two broad peaks. There is a good quantitative correlation for real part Re($\varepsilon_{1}$) between our 4aSi data (supercell dimension is 1.536 nm) and 3 nm result of Reference [19] (see Figure 9a). The quantitative correlation for Im($\varepsilon_{1}$) is observed below ∼3.5 eV, showing only a similar qualitative energy dependence above 3.5 eV. As seen in Figure 9, Re($\varepsilon_{1}$) and Im($\varepsilon_{1}$) of 6 nm sized Si thin films manifest an intermediate energy dispersion between two marginal cases, 3 nm and 12 nm. The 6 nm Si supercell was not considered in our study. Summarizing this comparison with Petrik et al.’s data, one may draw the following conclusion. First-principles calculation of dielectric properties of nano-porous Si feels the dimensionality effects up to ∼10 nm supercell. Larger Si supercells give the dielectric response similar to the bulk Si modification. Smallest Si supercells of an order of ∼1.5 to 3 nm, demonstrate practically the same dielectric response below 4 eV irrespective of the calculation method, that is, *ab initio* or MDF model.

**Figure 10:**
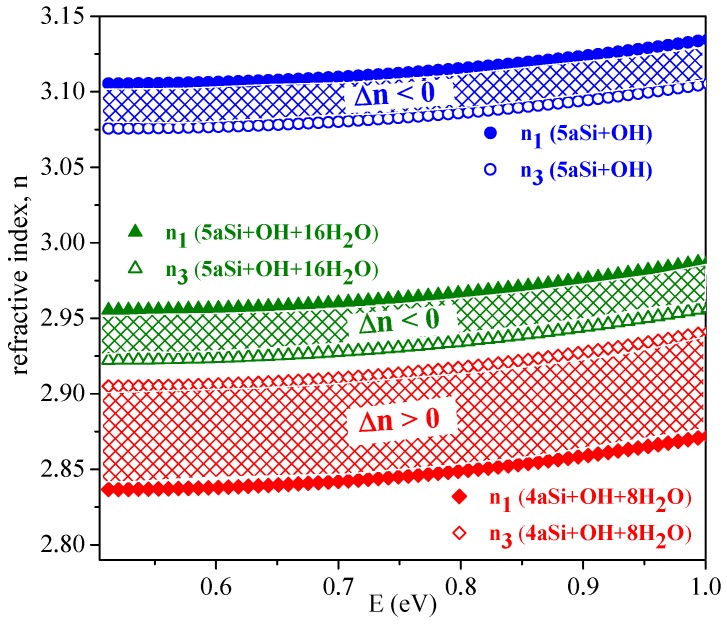
Refractive indices, n${}_{1}$ and n${}_{3},$ and respective birefringence Δn of two supercells, 4aSi and 5aSi, filled with hydroxyl groups and water molecules. All full symbols correspond to n${}_{1}$ index whereas all empty symbols correspond to n${}_{3}$ index.

Optical anisotropy of nanoporous Si composites in the optical transparency region (E < 1 eV) deserves a special discussion. Isotropic media with uniaxially aligned cylindrical nanopores are characterized by optical anisotropy [57] also referred to as geometric birefringence Δn. Figure 10 shows the calculated refractive indices and respective Δn in the optical transparency region for two porous Si supercells, 4aSi and 5aSi, decorated with hydroxyl or hydroxyl-water surface layers, that is, 4aSi+OH+8H${}_{2}$O, 5aSi+OH and 5aSi+OH+16H${}_{2}$O. For pure porous Si of porosity P = 0.25, that is, for the same porosity as in the case of our 4aSi structure, the generalized Bruggeman equation (for evaluation methodology see Reference [57]) gives the refractive indices n${}_{1}$ = 2.753 and n${}_{3}$ = 3.035 and thus a positive birefringence (Δn = n${}_{3}$ − n${}_{1}>$ 0). Note that the effective medium approach provides here a positive birefringence in the entire range of porosities (0 < P < 1). Our *ab initio* calculations, by contrast, predict a weak but negative birefringence, Δn < 0, for porous Si with hydroxyl 5aSi+OH or hydroxyl-water surface layers 5aSi+OH+16H${}_{2}$O, see Figure 10. Probably, such a difference of optical properties may be attributed to the strong optical anisotropy of the ordered OH and OH+H${}_{2}$O layers which may result in a considerable negative contribution to the optical birefringence. In other words, the effective (averaged) birefringence, Δn, may be represented as superposition of the positive geometrical birefringence of the porous Si matrix alone, Δn${}_{Si}$ > 0, and the negative birefringence of the anisotropic hydroxyl or hydroxyl-water layer(s) covering the pore walls, Δn${}_{OH+H_{2}O}$ < 0. *Ab initio* simulation shows that the molecular arrangement in the “adsorbed” OH and H${}_{2}$O layers should play a crucial role in the effective optic anisotropy of nanoporous Si+OH+H${}_{2}$ composite materials. A prominent example here may be the 4aSi+OH+8H${}_{2}$O composite, which in contrast to the 5aSi+OH and 5aSi+OH+16H${}_{2}$O ones, is characterized by a weak but positive birefringence. Apparently, this means that Δn${}_{Si}$ + Δn${}_{OH+H_{2}O}$ = Δn < 0 for 5aSi+OH and 5aSi+OH+16H${}_{2}$O whereas Δn${}_{Si}$ + Δn${}_{OH+H_{2}O}$ = Δn > 0 for 4aSi+OH+8H${}_{2}$O. Such a finding demonstrates the importance of OH and H${}_{2}$O arrangement in pore surface layer(s) for the optical anisotropy of nanoporous materials and may be of interest in nanoengineering of photonic materials.

## 6. Conclusions

We performed a detailed computational study of porous silicon filled with water molecules. We established the impact of nanoconfinement on the water structuration and hydrogen bonding within the pores of 7.34, 11.26 and 15.40 Å. Hydroxyl OH groups confined within the Si pores of 7.34 Å diameter appear to be disintegrated, thus forming free non-bonded protons which might be the reason for proton conductivity. However, the embedding of water molecules in these pores of the smallest diameter destroys the proton conductivity, thus retrieving the small but distinct energy gap in the electronic spectrum.

Energy band-gap of porous Si filled with water molecules increases with an increasing pore diameter according to nearly linear law. According to our calculations, the energy band-gap of bulk and porous Si of small diameter d ⩽ 11.26 Å (4aSi and 5aSi supercells) is of *indirect* type and the water filling of the Si pores does not transform the type of E${}_{ind}$. However, with an increase of the pore diameter to 15.40 Å (6aSi supercell), the *indirect* band-gap E${}_{ind}$ changes to the *direct* E${}_{dir}$ one. Embedding the water to 6aSi supercell enlarges the E${}_{dir}$ band-gap.

The calculated dispersion of refractive indices and the extinction coefficient of bulk Si nicely agree with the experimental data proving the reliability of our calculations. Si porosity augments the optical anisotropy ($n_{1}$ > $n_{3}$), especially in ultraviolet region and enlarges the extinction coefficients *k* of porous Si in the visible spectral range.

We described the structural evolution of water molecules and hydrogen bonds at an increased Si pore diameter and found that the geometry of hydrogen bonds deviates from the typical values due to the space confinement in the smallest 7.34 Å pores but tends to the normal values with enlarging the pore diameter to 15.40 Å.

Optical properties of porous Si decorated with hydroxyl OH and water H${}_{2}$O layers strongly depend on Si porosity and on OH+H${}_{2}$O morphology inside the pores. The first-principles modeling, in this respect, may be considered as an efficient tool in the technology of nanomaterials, for preliminary evaluation, prediction and/or tailoring of their optical properties.

## Figures and Tables

**Table 1:** Crystalline and computational parameters of the bulk and nanoporous Si structures. n${}_{uc}$ is the number of atoms in the unit cell; n(H${}_{2}$O) corresponds to the number of water molecules deposited in the unit cell; E${}_{g}$ is the band-gap energy (in eV), notations *dir* and *ind* correspond to *direct* and *indirect* energy band-gaps, respectively; f${}_{max}$ is the maximal force acting on each atom (in eV/Å). Experimental value of E${}_{g}$ [45] was obtained at 302 K.

Type	n${}_{\mathrm{uc}}$	n(H${}_{2}$O)	E${}_{g}$	Grid	f${}_{\max}$
			eV		eV/Å
Si (bulk)
(experim.)			1.14
Si (bulk)	8		0.575 (ind)	4 * 4 * 2	2.44 × 10${}^{-7}$
4aSi+OH	67	-	-	4 * 4 * 2	4.64 × 10${}^{-6}$
4aSi+H${}_{2}$O	91	8	0.752 (ind)	4 * 4 * 2	8.95 × 10${}^{-6}$
5aSi+OH	103	-	0.799 (ind)	2 * 2 * 2	1.45 × 10${}^{-6}$
5aSi+H${}_{2}$O(1)	127	8	0.809 (ind)	2 * 2 * 2	1.32 × 10${}^{-5}$
5aSi+H${}_{2}$O(2)	151	16	0.766 (ind)	2 * 2 * 2	3.18 × 10${}^{-6}$
6aSi+OH	139	-	0.808 (dir)	2 * 2 * 2	1.27 × 10${}^{-4}$
6aSi+H${}_{2}$O	211	24	0.862 (dir)	2 * 2 * 2	9.77 × 10${}^{-6}$

**Table 2:** The calculated structural parameters of hydroxyl OH groups and water molecules H${}_{2}$O of nanoporous Si structures.

OH, H${}_{2}$O		Hydrogen Bonds				
**angle**	**w**	**Hydrogen**	**O-H**	**H** ··· **O**	*∠* **O-H** ··· **O**	**V** ${}_{H}$
	**(°)**	**Contact**	**(Å)**	**(Å)**	**(°)**	
4aSi+H${}_{2}$O						
H${}_{1}$-O${}_{1}$-H${}_{1}^{{}^{\prime }}$	99.4	O${}_{1}$-H${}_{1}$···O${}_{2}$	1.01	1.68	144.6	1.11
H${}_{2}$-O${}_{2}$-H${}_{2}^{{}^{\prime }}$	93.9	O${}_{3}$-H${}_{3}$···O${}_{2}$	1.02	1.58	148.8	1.02
		O${}_{2}$-H${}_{2}$···O${}_{2}$		1.19	175.7	1.07
5aSi+OH						
Si${}_{15}$-O${}_{2}$-H${}_{2}$	118.1	O${}_{2}$-H${}_{2}$···O${}_{1}$	0.97	2.16	166.7	0.80
Si${}_{16}$-O${}_{3}$-H${}_{3}$	116.1					
5aSi+H${}_{2}$O(1)						
H${}_{1}$-O${}_{1}$-H${}_{1}^{{}^{\prime }}$	102.3	O${}_{1}$-H${}_{1}$···O${}_{2}$	0.98	1.92	142.3	0.99
H${}_{2}$-O${}_{2}$-H${}_{2}^{{}^{\prime }}$	108.7	O${}_{2}$-H${}_{2}$···O${}_{4}$	0.97	2.29	147.8	0.93
Si${}_{16}$-O${}_{3}$-H${}_{3}$	109.5	O${}_{3}$-H${}_{3}$···O${}_{1}$	1.03	1.58	176.6	1.06
Si${}_{15}$-O${}_{4}$-H${}_{4}$	114.8	O${}_{4}$-H${}_{4}$···O${}_{5}$	0.98	1.94	177.0	0.91
5aSi+H${}_{2}$O(2)						
H${}_{1}$-O${}_{1}$-H${}_{1}^{{}^{\prime }}$	105.5	O${}_{1}$-H${}_{1}$···O${}_{4}$	0.97	1.83	144.3	1.07
H${}_{2}$-O${}_{2}$-H${}_{2}^{{}^{\prime }}$	105.6	O${}_{2}$-H${}_{2}$···O${}_{3}$	1.01	1.57	176.6	1.10
H${}_{3}$-O${}_{3}$-H${}_{3}^{{}^{\prime }}$	99.8	O${}_{3}$-H${}_{3}$···O${}_{6}$	0.98	1.75	147.5	1.14
H${}_{4}$-O${}_{4}$-H${}_{4}^{{}^{\prime }}$	107.6	O${}_{4}$-H${}_{4}$···O${}_{6}$	0.97	1.89	140.8	1.06
Si${}_{16}$-O${}_{5}$-H${}_{5}$	120.1	O${}_{5}$-H${}_{5}$···O${}_{1}$	0.99	1.72	178.8	1.08
Si${}_{15}$-O${}_{6}$-H${}_{6}$	112.9	O${}_{6}$-H${}_{6}$···O${}_{7}$	0.98	1.82	154.4	0.98
6aSi+OH						
Si${}_{17}$-O${}_{2}$-H${}_{2}$	115.7	O${}_{2}$-H${}_{2}$···O${}_{1}$	0.97	2.16	166.7	0.87
Si${}_{18}$-O${}_{3}$-H${}_{3}$	118.0	O${}_{3}$-H${}_{3}$···O${}_{2}$	0.98	1.88	164.6	0.95
Si${}_{19}$-O${}_{4}$-H${}_{4}$	117.6					
6aSi+H${}_{2}$O						
H${}_{1}$-O${}_{1}$-H${}_{1}^{{}^{\prime }}$	102.8	O${}_{1}$-H${}_{1}$···O${}_{3}$	0.98	1.83	167.1	0.98
H${}_{21}$-O${}_{2}$-H${}_{22}$	111.4	O${}_{2}$-H${}_{21}$···O${}_{3}$	0.99	1.63	163.0	1.00
		O${}_{2}$-H${}_{22}$···O${}_{1}$	0.98	1.91	151.1	0.98
H${}_{31}$-O${}_{3}$-H${}_{32}$	105.8	O${}_{3}$-H${}_{31}$···O${}_{7}$	0.99	1.77	158.1	1.07
		O${}_{3}$-H${}_{32}$···O${}_{4}$	0.98	1.92	141.7	1.04
H${}_{4}$-O${}_{4}$-H${}_{4}^{{}^{\prime }}$	105.0	O${}_{4}$-H${}_{4}$···O${}_{6}$	0.98	1.92	148.1	1.05
Si${}_{17}$-O${}_{6}$-H${}_{6}$	119.0	O${}_{6}$-H${}_{6}$···O${}_{3}$	0.97	2.12	137.8	0.94
Si${}_{18}$-O${}_{7}$-H${}_{7}$	114.3	O${}_{7}$-H${}_{7}$···O${}_{2}$	1.01	1.64	167.6	1.04
Si${}_{19}$-O${}_{8}$-H${}_{8}$	110.8	O${}_{8}$-H${}_{8}$···O${}_{2}$	0.97	2.55	138.7	0.90
